# Structural Racism and Triple-Negative Breast Cancer Among Black and White Women in the United States

**DOI:** 10.1089/heq.2021.0041

**Published:** 2022-02-14

**Authors:** Linsey Eldridge, David Berrigan

**Affiliations:** ^1^ National Cancer Institute, Center for Global Health, Rockville, Maryland, USA.; ^2^ National Cancer Institute, Division of Cancer Control and Population Sciences, Rockville, Maryland, USA.

**Keywords:** breast cancer, TNBC, structural racism, health disparities

## Abstract

**Purpose:** To determine the associations between state-level indicators of structural racism and incidence of triple-negative breast cancer (TNBC) among black and white women diagnosed with breast cancer.

**Methods:** Black and white women diagnosed with breast cancer between 2010 and 2016 were identified from 12 states represented in the Surveillance, Epidemiology, and End Results (SEER18) program. State-level disparities were measured by black to white rate ratios in educational attainment, political participation, incarceration, and unemployment; and dichotomized to “high” or “low” structural racism using the median rate ratio of the 12 states. Logistic regression was used to examine the associations between indicators of structural racism and TNBC among black and white women.

**Results:** Living in states with high levels of structural racism in the domains of educational attainment, judicial treatment, and political participation were generally associated with greater odds of TNBC among black and white women. The increased odds of TNBC was greater for black women living in states with high levels of racial disparities than white women. Among black women diagnosed with breast cancer, the odds ratio (OR) of being diagnosed with TNBC comparing women living in states with high disparities in educational attainment versus those with low disparities was 1.50 (95% confidence interval [CI]: 1.27–1.77). For white women, the OR for educational attainment was 1.17 (95% CI: 1.10–1.23).

**Conclusion:** Results from this study support the notion that racial health disparities need to be contextualized. Further research should address mechanisms through which structural racism influences health disparities.

## Introduction

There is an important body of literature seeking to understand the role of racism in health disparities. Many of these studies addressing the impact of racism on health rely on self-reported measures of interpersonal discrimination.^1,2^ In contrast, structural racism refers to racism at the macro-level and recognizes the ways systems such as housing, education, and employment interact to produce racialized outcomes.^3^ Documenting individual experiences of discrimination is important; however, understanding the ways racism functions at the structural level to influence health necessitates further investigation.

Interest in understanding structural racism is growing; however, limitations to measures of structural racism remain a hindrance to fully studying its effect on health.^4–6^ A systematic review of studies conducted in the past 10 years involving health and structural racism found that most measures were within the neighborhood, focusing primarily on residential segregation.^7^ Furthermore, the number of studies examining the link between structural racism and cancer risk is sparse. Groos et al.'s systematic review lists a single study addressing cancer, despite gaping disparities in cancer incidence and mortality.^7^ That study found that black women living in neighborhoods characterized by racial bias in mortgage lending experienced poorer colorectal cancer outcomes.^8^

A more recent study also utilizing neighborhood-level measures of structural racism revealed that living in redlined census tracts was associated with an increased risk of breast cancer mortality for both non-Hispanic black and white women.^9^ Although neighborhood-level measures of structural racism play an important role in health, they offer a limited perspective on the complex systems that embody structural racism.^10–12^

In another study, Krieger et al. utilize state-level policies as a measure of structural racism, finding that birthplace in a state with a history of Jim Crow ordinances was associated with an increased risk of estrogen receptor negative (ER^−^) breast cancer subtypes among black, but not white women.^13^ Krieger et al. selected ER^−^ for attention, citing evidence that breast cancer subtype may be affected by early life, adolescent, and adult exposures.^14^

Both ER^−^ tumors, that account for <30% of breast cancer cases, and triple-negative breast cancer (TNBC), characterizing 10–15% of breast cancer cases, are significant sources of disparities in breast cancer outcomes and have important clinical implications. TNBC is defined as cases where cancer cells lack joint hormone receptor (HR; estrogen [ER] and progesterone receptors [PR]) and display low levels of human epidermal growth factor receptor (HER2).^15^ Black women are approximately twice as likely to be diagnosed with TNBC than white women.^16^ TNBC is associated with larger and higher-grade carcinomas at diagnosis and cannot be treated by endocrine-based treatments,^17^ resulting in higher rates of 5-year cancer-related mortality compared with other types of breast cancer (40% vs. 20%).^18^

Incidence of TNBC and the strength of the racial disparity vary by geographic region, suggesting environmental and social factors may be associated with the subtype; however, these factors remain largely understudied.^19,20^ Instead, studies on breast cancer subtype continue to focus on individual and genetic factors.^21^ Although the Duffy-null allele, a West African ancestral variant, is known to be associated with TNBC,^22^ relying solely on genetics neglects the environmental and social exposures that disproportionately affect people by race.

Furthermore, the racial disparity of TNBC incidence varies by state, which cannot be explained by genetics, as there is no evidence to suggest African ancestry among black Americans also varies significantly by state.^23^ In addition, evidence indicates that expression of ER is regulated by methylation and is modifiable,^17,24–27^ further supporting exploration of behavioral and environmental factors influencing racial disparities.

Individual-level factors such as diet and physical inactivity, known to be associated with TNBC,^28,29^ may be mediators on the pathway from community-level factors to clinical presentations of breast cancer; however, they do not represent the fundamental causes of health and health disparities.^30^ Limited access to fresh fruits and vegetables in minority neighborhoods^21^ and inequitable distribution of recreational resources in primarily black neighborhoods^31^ may contribute to diet and physical inactivity. Furthermore, a recent study on the association between body mass index (BMI) and county-level indicators of structural racism found that high levels of racism was associated with a higher BMI in black, but a lower BMI in white people. This finding was stronger for black women than black men.^4^

Addressing breast cancer subtype disparities requires moving beyond genetic and individual factors, and toward addressing the social context that shapes health. Building on previous work showing state-level variation in policies and laws resulted in differential health outcomes for sexual minorities,^32,33^ Lukachko et al. examined the ways state-level variation in political participation, employment, education, and incarceration predict myocardial infarction (MI). Results from this study indicated that living in states with high exposure to structural racism in these domains was associated with increased prevalence of MI for black, but not white people, regardless of individual age, sex, education, income, and medical insurance status.^2^

This study builds on Lukachko et al.'s work and addresses some of the aforementioned limitations of previous research on TNBC disparities by examining state-level variation in measures of structural racism across the domains of political participation, employment, educational attainment, and incarceration to assess whether racism differentially predicts TNBC risk among black and white women in the United States.

## Methods

### Data source

Non-Hispanic black and white women diagnosed with breast cancer between January 1, 2010 and December 31, 2016 were identified from 17 population-based cancer registries that participate in the SEER program.^34^ Only cases diagnosed after 2010 were included because this is when SEER began collecting HER2 receptor status, used to define breast cancer subtype. The 12 states represented in the data set were California, Connecticut, Georgia, Hawaii, Iowa, Kentucky, Louisiana, Michigan, New Mexico, New Jersey, Washington, and Utah. The analytical data set included 301,600 non-Hispanic white women and 46,853 non-Hispanic black women. A full IRB review was not required, because the NIH's Office of Human Subjects Research have determined that the SEER data are exempt (CFR 46.104(4)).

### Measures of structural racism

Measures of structural racism comprised four domains: educational attainment, employment, judicial treatment, and political participation. State-level disparities across these domains were identified by Lukachko et al. as representing the systematic exclusion of black people from resources and societal mobility.^2^

The domain of *educational attainment* was the relative proportion of blacks to whites over 15 years of age with a bachelor's level degree or higher. These statistics were derived from the U.S. Census Bureau, Decennial Census, 2010 (https://www.census.gov/data/tables/2010/demo/educational-attainment/cps-detailed-tables.html).^35^

*Employment* was the relative state-level unemployment rate ratio between blacks and whites. These data were obtained from the U.S. Bureau of Labor Statistics, 2010 (https://www.bls.gov/opub/ted/2011/ted_20111005.htm?view_full).^36^

*Judicial treatment* included two measures: (1) relative proportion of blacks to whites incarcerated (jails and prisons) and (2) relative proportion of blacks to non-blacks among the voting age population disenfranchised due to felony convictions. Felony disenfranchisement rates for whites for 2010 were not available. Incarceration and disenfranchisement data were derived from the U.S. Department of Justice, 2010 by Sakala.^37,38^

Lastly, *political participation* also included two measures: (1) relative proportion of blacks to whites 18 years of age and over in each state who were registered to vote in 2010 and (2) relative proportion of blacks to whites 18 years of age and over who voted in 2010. Data were obtained from the U.S. Census Bureau, Decennial Census 2010 (https://www.census.gov/data/tables/2010/demo/voting-and-registration/voting-registration-2010-election.html).^39^

Relative rate ratios of blacks to whites in each domain were calculated for each of the 12 states. The median rate ratio for each domain was used as a cut-point to dichotomize each state-level measure of structural racism to indicate low versus high exposure to structural discrimination. The median was used as a cut-point for consistency and ease of interpretation.

### Breast cancer subtype

Breast cancer subtypes are defined by joint hormone receptor (HR and PR) and HER2 status. Using subtype information, tumors were classified into five categories: (1) HER2^+^/HR^+^, (2) HER2^+^/HR^−^, (3) HER2^−^/HR^+^, (4) ER^−^/PR^−^/HER2^−^ (triple negative), or (5) unknown.^34^ For this study, TNBC status was dichotomized using the aforementioned five categories. The first three categories were classified as non-TNBC and the fourth category was classified as TNBC. Cases with unknown subtype were not included in this study.

### Covariates

Demographic characteristics, including age and marital status, were ascertained across SEER registries using standardized coding methods based on hospital medical records.^40^ Analyses were adjusted for individual age and marital status because both were associated with TNBC diagnosis (Table 1), but we were unable to examine individual education and socioeconomic status because they are not provided by SEER registries.

**Table 1.** Distribution of Clinical, Demographic, and State-Level Indicators of Structural Racism by HER2^−^/HR^−^ (TNBC) Status, SEER18 Registry 2010–2016

	White women *N* (%) or mean (std. dev.)			Black women *N* (%) or mean (std. dev.)		
	Non-TNBC *N*=297,614 (90.0%)	TNBC *N*=3986 (10.0%)	*p*	Non-TNBC *N*=37,398 (78.8%)	TNBC *N*=9455 (20.2%)	*p*
Age	62.7 (13.3)	60.0 (14.3)	<0.0001^a^	59.9 (13.04)	57.4 (13.06)	<0.0001^a^
Marital status			0.0008^b^			0.0010^b^
Never married	38,225 (13.6%)	4454 (14.2%)		11,119 (31.7%)	2982 (33.5%)	
Been married	243,741 (86.4%)	26,826 (85.8%)		23,990 (68.3%)	5921 (66.5%)	
Grade			<0.0001^b^			<0.0001^b^
Grade I well differentiated	76,125 (25.6%)	746 (2.3%)		6479 (17.3%)	145 (1.5%)	
Grade II: moderately differentiated	137,354 (46.1%)	5897 (17.9%)		15,822 (42.3%)	1328 (14.0%)	
Grade III: poorly differentiated	69,916 (23.5%)	24,370 (73.9%)		12,647 (33.8%)	7437 (78.7%)	
Grade IV: undifferentiated	785 (0.3%)	283 (0.9%)		90 (0.2%)	58 (0.6%)	
Unknown	13,434 (4.5%)	1690 (5.1%)		2360 (6.3%)	487 (5.1%)	
State-level disparity						
Education			<0.0001^b^			0.8030
Low	160,665 (54.0%)	18,340 (55.6%)		14,037 (37.5%)	3562 (37.7%)	
High	136,949 (46.0%)	14,646 (44.4%)		23,361 (62.5%)	5893 (62.3%)	
Unemployment			0.0052^b^			0.4507
Low	195,217 (65.6%)	21,891 (66.4%)		20,145 (53.9%)	5134 (54.3%)	
High	102,397 (34.4%)	11,095 (33.6%)		17,253 (46.1%)	4321 (45.7%)	
Incarceration			0.1264			0.9346
Low	90,660 (30.5%)	10,183 (30.9%)		16,907 (45.2%)	4270 (45.2%)	
High	206,954 (69.5%)	22,803 (69.1%)		20,491 (54.8%)	5185 (54.8%)	
Disenfranchisement			0.1978			0.0102
Low	86,144 (28.9%)	9436 (28.6%)		17,204 (46.0%)	4489 (47.5%)	
High	211,470 (71.1%)	23,550 (71.4%)		20,194 (54.0%)	4966 (52.5%)	
Voter registration						0.5328
Low	121,371 (40.8%)	13,411 (40.7%)	0.6619	23,571 (63.0%)	5992 (63.8%)	
High	176,243 (59.2%)	19,575 (59.3%)		13,827 (37.0%)	3463 (36.63%)	
Voting			0.0016^b^			0.0097^b^
Low	109,051 (36.6%)	11,795 (35.8%)		26,310 (70.3%)	6780 (71.7%)	
High	188,563 (63.4%)	21,191 (64.2%)		11,088 (29.6%)	2675 (28.3%)	

^a^ Student's *t*-test.

^b^ Chi-square test.

HER2, human epidermal growth factor receptor; SEER18, Surveillance, Epidemiology, and End Results; TNBC, triple-negative breast cancer.

### Statistical analysis

State-level indicators of structural racism were linked to individual-level data from SEER using federal information processing standards codes. Patients were assigned a value for state-level indicators based on the state of residence at time of diagnosis to represent high or low exposure to structural discrimination. Logistic regression analyses were used to measure the association between indicators of structural racism and TNBC status by race. The interaction between race and indicators of structural racism in predicting TNBC using multiplicative interaction terms in the model was also assessed. All models were adjusted for age and marital status. All statistical analysis was done using SAS 9.4 statistical software.

## Results

### Distribution of measures of structural racism

Table 2 shows the distribution of measures of structural racism by race. The median for the ratio measures representing educational attainment and political participation were below 1.0, meaning black people were under-represented in these domains relative to whites. For these two domains, states with a rate ratio greater than the median were classified as low exposure to structural racism, and a rate ratio equal to or less than the median were classified as high exposure to structural racism. For example, on average, 17% of black people and 26% of white people had a bachelor's degree or higher resulting in a median rate ratio of 0.59. Therefore, states with an educational attainment rate ratio of 0.59 or lower were classified as high exposure to structural discrimination.

**Table 2.** Distribution of State-Level Measures of Structural Racism

Measure of structural racism	Mean for black (SE)	Mean for white (SE)	Median ratios^a^ (SE)	Rate ratio range for states
Educational attainment				
% with bachelor's degree or higher	0.17 (0.02)	0.26 (0.01)	0.59 (0.07)	0.34–1.05
Employment				
Unemployment rate	16.51 (1.25)	8.42 (0.51)	2.04 (0.10)	1.41–2.41
Judicial treatment				
Incarceration rate	2.91 (0.48)	0.45 (0.05)	6.38 (0.84)	2.50–11.48
% disenfranchised	0.06 (0.02)	0.01 (0.005)^b^	5.04 (0.65)	1.86–8.92
Political participation				
% registered to vote	0.56 (0.05)	0.68 (0.02)	0.85 (0.07)	0.24–1.05
% voted	0.40 (0.02)	0.50 (0.02)	0.80 (0.05)	0.55–1.07

^a^ Rate ratio refer to relative proportions of blacks to whites within each state for the year 2010. Only the ratios from the 12 states represented in the SEER18 registry are included.

^b^ Rate of non-black disenfranchisement.

For measures of unemployment and judicial treatment, the median rate ratios were above 1.0, meaning black people were over-represented in these domains. Therefore, states with a rate ratio at the median or above were classified as high exposure to structural discrimination.

### Distribution of demographic, clinical, and state-level indicators of structural racism by TNBC status and race

Table 1 shows the distribution of clinical, demographic, and state-level indicators of structural racism by TNBC status and race. White and black women diagnosed with TNBC were significantly younger (60 and 57.4, respectively), than women diagnosed with all other subtypes (62.7 and 59.9, respectively). Marital status was also significantly associated with TNBC status for both white and black women. White and black women diagnosed with TNBC were significantly more likely to be diagnosed at grade III (73.9% and 78.7%) than women diagnosed with all other subtypes.

### Structural racism and TNBC among black women

As shown in Table 3, in models adjusting for age and marital status, associations were statistically significant for disparities in attainment of a bachelor's degree or higher (odds ratio [OR]=1.50; 95% confidence interval [CI]: 1.27–1.77), disparities in disenfranchisement rates (OR=1.27; 95% CI: 1.17–1.38), and disparities in voting practices (OR=1.33; 95% CI: 1.21–1.45). These results indicate that black women living in states with high levels of disparities in these areas were more likely to be diagnosed with TNBC than black women living in states with low levels of disparities in these areas.

**Table 3.** OR and 95% CI for HER2^−^/HR^−^ (TNBC) Among Non-Hispanic Black and Non-Hispanic White Women Diagnosed with Breast Cancer Between 2010 and 2016 (18 SEER Registry Group), in Relation to High Levels of Structural Racism in Four Domains

Measure of structural racism^a^	HER2^−^/HR^−b^				Interaction between race and measures of structural racism	
	Black		White			
	OR (95% CI)	*p*	OR (95% CI)	*p*	Wald chi-square	*p*
Educational attainment						
Bachelor's degree or higher	1.50 (1.27–1.77)	<0.0001	1.17 (1.10–1.23)	<0.0001	60.75	<0.0001
Employment						
Unemployment rate	1.07 (0.95–1.21)	0.2793	0.99 (0.95–1.04)	0.8123	6.10	0.0135
Judicial treatment						
Incarceration	0.89 (0.79–1.01)	0.0689	0.99 (0.95–1.03)	0.6472	1.92	0.1661
Disenfranchisement	1.27 (1.17–1.38)	<0.0001	1.10 (1.05–1.16)	<0.0001	38.78	<0.0001
Political participation						
Voter registration	1.05 (0.92–1.20)	0.4819	1.07 (1.03–1.12)	0.0009	2.90	0.0883
Voting practices	1.33 (1.21–1.45)	<0.0001	1.00 (0.96–1.04)	0.9086	117.74	<0.0001

^a^ Relative proportion of blacks to whites in domain.

^b^ Adjusted for age and marital status.

CI, confidence interval; OR, odds ratio.

### Structural racism and TNBC among white women

High levels of structural racism across the domains of educational attainment, judicial treatment, and political participation were generally associated with greater odds of being diagnosed with TNBC among white women diagnosed with breast cancer. In models adjusted for age and marital status, associations were statistically significant for disparities in attainment of a bachelor's degree or higher (OR=1.17; 95% CI: 1.10–1.23), disparities in disenfranchisement (OR=1.10; 95% CI: 1.05–1.16), and disparities in voter registration (OR=1.07; 95% CI: 1.03–1.12). These results indicate that white women living in states with high disparities in these areas were more likely to be diagnosed with TNBC than white women living in states with states with low disparities.

### Interactions between structural racism and race

The associations between indicators of structural racism and TNBC significantly differed by race across most domains (Table 3). Interactions between structural racism and race were statistically significant for measures of educational attainment (*p*<0.0001), employment (*p*=0.0135), disenfranchisement (*p*<0.0001), and voting practices (*p*<0.0001). These results indicate that the effect of these measures on TNBC diagnosis differs significantly among black and white women. Specifically, although the association between disparities in the areas of educational attainment and disenfranchisement and TNBC were positive among both black and white women, the associations were significantly weaker for white women.

In contrast, whereas the association between the disparity in voting practices and TNBC were positive among black women (resulting in higher prevalence of TNBC), they were null among white women (*p*<0.0001). Finally, although the interaction between race and disparities in unemployment was statistically significant (*p*=0.0135), the ORs were not significant for either black or white women, suggesting that this interaction is likely not practically relevant.

## Discussion

This study analyzed data on breast cancer subtype for patients from the Surveillance, Epidemiology, and End Results (SEER18) database to demonstrate the difference in the occurrence of TNBC by state-level measures of structural racism. The measures used move beyond established neighborhood-level measures of structural inequities to examine the ways in which judicial treatment, employment, education, and political participation impact breast cancer.

In states that measured higher in indicators of structural racism the incidence of TNBC was greater in both black and white women. Furthermore, for three of the four measures, the increase in TNBC was higher in black than white women. These findings support the hypothesis that living in states with high measures of structural racism increases the odds of TNBC diagnosis among black women diagnosed with breast cancer.

The associations between TNBC and structural racism observed in this study were not specific to black women, and among white women diagnosed with breast cancer, state-level indicators of structural racism also appear to increase the odds of TNBC. This finding was unexpected as other studies have found that measures of structural racism are protective for whites.^2,4,13^ However, these studies used either different measures of structural racism or a different health outcome, which may explain these inconsistences.

There are at least two possible explanations for why racial disparities increase the odds of TNBC for white women. First, the factors that mediate the relationship between structural racism and TNBC for black women may also affect TNBC for white women. For example, racial disparities in disenfranchisement rates were associated with an increased odd of TNBC for both black and white women. The process by which disenfranchisement disparities affect health may include differential allocation of health resources as a result of inequitable public policies.^41^

Studies exploring the link between voter disenfranchisement and health disparities suggest that states with low disparities in felon disenfranchisement are more likely to have more equitable public policies that improve health for both white and black people.^42,43^ In this scenario, structural racism *per se* is not a causal factor influencing incidence of TNBC in white women, rather it is an indicator variable for state-level characteristics of health systems that influence TNBC incidence.

A second possible explanation is that states with high racial disparities may also have high gender disparities that negatively impact white women. Although the increased odds of TNBC was not specific to black women, the strength of the disparity differed by race. The excess odds of TNBC was greater for black women living in states with high levels of racial disparities than white women living in the same states. These findings indicate that state-level measures of structural racism deserve investigation as a social determinant of breast cancer subtype disparities. Although racism affects all states, the measures of discrimination were greater in states that measured “high” in structural racism, thus potentially increasing the risk of TNBC.

Differences in state-level policies in education, judicial treatment, political participation, and employment produce varying degrees of inequities and have direct implications on health. This is supported by other studies where exposure to high levels of structural racism was shown to increase the risk of various health issues, including MI and ER^−^ breast cancer.^2,13,44^ State-level policies have the potential to increase inequities or protect marginalized groups. Further attention to the geography of racial disparities is also warranted, the optimal spatial scale for policies to address structural racism and its consequences is not completely understood.

The study has several limitations. First, analysis was based solely on the 12 states in the SEER18 database and would have benefitted from inclusion of a greater number of states. Although SEER18 is thought to be representative of the U.S. population in terms of race and age, measures of structural racism may not be fully represented by the 12 states in this study. Second, SEER18 does not include individual-level variables that could confound the association between structural racism and TNBC, including SES, obesity, and breastfeeding rates, among others. Future studies should employ a multilevel analysis to adjust for these variables. Third, patients were assigned to exposure levels based on state of residence at time of diagnosis, not the state they were born and/or lived in the longest.

Despite these limitations, this study has several strengths. First, this is the first study we are aware of to use these measures of structural racism to examine disparities in TNBC, adding to the literature examining health outcomes and structural racism. To our knowledge, only three prior studies have examined associations between structural racism and cancer incidence or mortality.^8,13,45^ However, a larger number of studies have linked structural racism to other behavioral, physiological, and disease outcomes.^4,46,47^

Second, although this was the first study to use these measures of structural racism and TNBC, these measures have been examined in previous studies of cardiovascular disease.^2^ Third, SEER18 covered about 28% of the U.S. population in 2010 and is generally thought to be representation of the United States.

## Conclusion

This study addresses some of the gaps in existing literature of discrimination and health disparities by utilizing innovative indicators identified by Lukachko et al.^2^ Furthermore, results from this study provide evidence of the impact of structural racism on breast cancer subtype. The limitations noted earlier can and should be addressed by future studies examining state-level indicators of structural racism and breast cancer subtype.

## Abbreviations Used

BMI: body mass index
CI: confidence interval
ER^−^: estrogen receptor negative
HER2: human epidermal growth factor receptor
HR: hormone receptor
MI: myocardial infarction
OR: odds ratio
PR: progesterone receptor
SEER18: Surveillance, Epidemiology, and End Results
TNBC: triple-negative breast cancer
